# 

**Published:** 1848

**Authors:** 


### BRITISH AND FOREIGN
### MEDICAL REVIEW

OR

### QUARTERLY JOURNAL

OF

### PRACTICAL MEDICINE AND SURGERY

---

EDITED BY

JOHN FORBES M.D. F.R.S. F.G.S.

---

SURGEON GEN'L'S OFFICE

182963

LIBRARY

VOL. XXV.

BEING

### A GENERAL INDEX

TO THE PRECEDING

TWENTY-FOUR VOLUMES

LONDON
JOHN CHURCHILL PRINCES STREET SOHO
MDCCCXLVIII.

W1
BR34

C. AND J. ADLARD, PRINTERS, BARTHOLOMEW CLOSE.

33757

### ADVERTISEMENT.

The Editor of the British and Foreign Medical Review is at length enabled, by the publication of the present volume, to fulfil the promise made to his subscribers in the Postscript to the Forty-eighth Number, and thus to complete and to render as perfect as it is in his power to make it, the work on which he has been so long engaged. Although this Index is not so minute as it might have been made, it is believed that it contains every reference requisite for the complete examination of the contents of the work to which it is appended. Such examination, it is hoped, will be not a little facilitated by the various subjects being referred to under as many and various heads as was found practicable, as well as by the conspicuous manner in which all the references are given—each reference having its own separate line, with the volume and page conspicuously displayed in one invariable order of sequence and position. It will be observed that the alphabetical arrangement is carried out to an unusual extent; all the subordinate references under each separate heading being placed in strict alphabetical order also. All the Authors' Names, except when occurring in the subordinate references, are printed in Capitals: by this arrangement, the necessity of having a separate List of Authors is avoided, as the difference of type will attract the immediate notice of such readers as consult the Index in search of the WORKS of particular writers. On the whole, it is hoped that the Index will be found not unworthy of the volumes of which it is the key.

For the construction of this very laborious work the Editor is entirely indebted to his friend, DR. ROBERT BOWER, on whose conscientious accuracy he has every reason to rely, and to whom he here offers his sincere acknowledgments and thanks.

J. F.

LONDON; *June* 30, 1848.

### DEDICATION.

### TO

FRANCIS ADAMS, LL.D., Member of the Royal Coll. of Surg., Edin., Banchory-Ternan,
W. P. ALISON, M.D., Professor of Medicine in the University of Edinburgh,
HENRY ANCELL, Esq., Surgeon to the Western General Dispensary, London,
JAMES ANDERSON, M.D., Inspector of Naval Hospitals, Haslar Hospital,
CHARLES ADLARD, Esq., London,
FRANCIS ARCHER, Esq., Surgeon to the Borough Prison, Liverpool,
NEIL ARNOTT, M.D., F.R.S., Physician Extraordinary to the Queen, London,
SAMUEL ASHWELL, M.D., Physician-Accoucheur to Guy's Hospital, London,
BENJAMIN GUY BABINGTON, M.D., F.R.S., Physician to Guy's Hospital, London,
T. GRAHAM BALFOUR, M.D., Grenadier Guards, London,
EDWARD BALLARD, M.D., Physician to the Royal St. Pancras Dispensary, London,
WILLIAM BALY, M.D., F.R.S., Physician to the Milbank Prison, London,
JAMES L. BARDSLEY, M.D., Consulting Phys. to the Royal Infirmary, Manchester,
GEORGE HILARO BARLOW, M.D., Physician to Guy's Hospital, London,
JOHN RHEA BARTON, M.D., Philadelphia, U. S.,
JAMES BEGBIE, M.D., Fellow of the Royal College of Physicians, Edinburgh,
WILLIAM BEILBY, M.D., Vice-President of the Royal Coll. of Physicians, Edinburgh,
WILLIAM BELL, M.D., Physician to the Metropolitan Convalescent Institution, London,
JAMES RISDON BENNETT, M.D., Lecturer on Materia Medica, and Assistant Physician at St. Thomas's Hospital, London,
ARCHIBALD BILLING, M.D., F.R.S., late Senior Physician to the London Hospital, and Examiner in Medicine at the University of London,
GOLDING BIRD, M.D., F.R.S., Lecturer on Materia Medica, and Assistant Physician at Guy's Hospital, London,
FRANCIS BOOTT, M.D., F.L.S., Member of the Council of University College, London,
HENRY BOWDITCH, M.D., Physician to the Massachusetts Hospital, Boston, U.S.,
JOHN BROWN, M.D., Fellow of the Royal College of Physicians, Edinburgh,
JOSEPH BROWN, M.D., Senior Physician to the Infirmary, Sunderland,
ISAAC BAKER BROWN, Esq., M.R.C.S., London,
W. A. F. BROWNE, M.D., Physician to the Asylum, Dumfries,
ALEXANDER BRYSON, M.D., R.N., Admiralty, London,
GEORGE BUDD, M.D., F.R.S., Professor of Medicine at King's College, London,
JOSEPH BULLAR, M.D., Surgeon to the Royal South Hants Infirmary, Southampton,
WILLIAM BULLAR, M.D., Surgeon to the Royal South Hants Infirmary, Southampton,
SIR WILLIAM BURNETT, K.C.H., M.D., F.R.S., Director-General of Naval Hospitals and Fleets,
EDWARD CALDWELL, Esq., Surgeon, R.N.,

LAWSON CAPE, M.D., late Lecturer on Midwifery at St. Thomas's Hospital, London,
RICHARD CARMICHAEL, Esq., F.R.S., M.R.I.A., Dublin,
WILLIAM B. CARPENTER, M.D., F.R.S., Examiner in Comparative Anatomy at the University of London,
ROBERT CARSWELL, M.D., Physician to H. M. the King of the Belgians, Brussels,
ROBERT CEELY, Esq., F.R.C.S., Surgeon to the Bucks Infirmary, Aylesbury,
EDWARD CHARLTON, M.D., Lecturer on Medicine at the Medical School, Newcastle-on-Tyne,
W. D. CHOWNE, M.D., Physician to the Charing-Cross Hospital, London,
JOHN CHURCHILL, Esq., London,
SIR JAMES CLARK, Bart., M.D., F.R.S., Physician in Ordinary to the Queen, London,
FREDERICK LE GROS CLARK, Esq., F.R.C.S., Assistant Surgeon to St. Thomas's Hospital, London,
WILLIAM CLARK, M.D., F.R.S., Professor of Anatomy in the University of Cambridge,
HENRY CLUTTERBUCK, M.D., formerly Lecturer on the Theory and Practice of Medicine, London,
JOHN COLDSTREAM, M.D., Fellow of the Royal College of Physicians, Edinburgh,
JOHN CONOLLY, M.D., F.R.C.P., Physician to the Hanwell Asylum, Hanwell,
J. T. CONQUEST, M.D., Physician to the London Lying-in Hospital, London,
THOMAS COPELAND, Esq., F.R.S., Surgeon Extraordinary to the Queen, London,
JAMES COPLAND, M.D., F.R.S., Consulting Physician to Queen Charlotte's Lying-In Hospital,
D. J. CORRIGAN, M.D., Physician to the Queen, Dublin,
JOHN COWPER, M.D., Professor of Materia Medica in the Uuiversity of Glasgow,
JAMES COWAN, LL.D., Dildawn,
CHARLES COWDELL, M.D., Oundle, Northamptonshire,
ABRAHAM COX, M.D., Kingston-on-Thames,
DAVID CRAIGIE, M.D., F.R.S.E., Fellow of the Royal Coll. of Physicians, Edinburgh,
SIR PHILIP CRAMPTON, Bart., F.R.S., Surgeon-General, Ireland,
W. E. CROWFOOT, Esq., F.R.C.S., Surgeon to the Beccles Dispensary, Beccles,
ANDREW CRAWFORD, M.D., Physician to the County Hospital, Winchester,
J. P. CROFT, M.D., University College, London,
C. P. CROKER, M.D., Dublin,
JOHN DALRYMPLE, F.R.C.S., Surgeon to the Royal Ophthalmic Hospital, London,
HENRY DANIEL, M.D., London,
ROBERT DAUN, M.D., Priory, Aberdeen,
HENRY DAVIES, M.D., Senior Physician to the British Lying-in Hospital, London,
GILMAN DAVIES, M.D., Portland, Maine, U. S.,
CHARLES DAVIS, M.D., late Professor of the Medical College, South Carolina, U. S.,
JOHN HALL DAVIS, M.D., Physician to the Royal Maternity Charity, London,
GEORGE E. DAY, M.D., Lecturer on Materia Medica at Middlesex Hospital,
DAVID DEAS, Esq., Surgeon, R.N.,
JOHN DRUMMOND, M.D., Deputy Inspector, R. N., Royal Marine Hospital, Woolwich,
JOHN JAMES DRYSDALE, M.D., Liverpool,
FREDERICK DUESBURY, M.D., Physician to the Invalid Asylum, Stoke-Newington,
W. H. DUNCAN, M.D., Physician to the Infirmary, Liverpool,
ROBLEY DUNGLISON, M.D., Professor of the Institutes of Medicine in Jefferson College, Philadelphia, U. S.,
ROBERT DYCE, M.D., Lecturer at Marischal College and University, Aberdeen,

G. VINER ELLIS, Esq., F.R.C.S., Demonstrator of Anatomy, Univ. College, London,
JOHN ERICHSEN, Esq., F.R.C.S., Lecturer on Physiology, Westm. Hospital, London,
J. B. ESTLIN, Esq., F.R.C.S., Surgeon to the Bristol Eye Dispensary, Bristol,
G. F. EVANS, M.D., Physician to the General Hospital, Birmingham,
WILLIAM FARR, Esq., M.R.C.S., General Register Office, Somerset House,
ROBERT FERGUSON, M.D., Physician-Accoucheur to the Queen, London,
WILLIAM FERGUSSON, Esq., F.R.S., Professor of Surgery, King's College, London,
JAMES CRAWFORD FERRIER, M.D., Brixton,
W. H. FISHER, M.D., Downing Professor of Medicine in the University of Cambridge,
ALEXANDER FLEMING, M.D., late President of the Royal Medical Society, Edinburgh,
CHARLES GALLAND, M.D., Professor of Anatomy, Malta,
ALFRED BARING GARROD, M.D., Assistant Physician to University College Hospital,
JOHN DURANCÉ GEORGE, Esq., Lecturer on Dental Surgery, Univ. College, London,
RICHARD F. GEORGE, Esq., F.R.C.S., Senior Surgeon, General Hospital, Bath,
T. W. GIBBON, M.D., H.E.I.C.S.,
W. GIBSON, M.D., Professor of Surgery, University of Pennsylvania, U.S.,
G. F. GIRDWOOD, M.D., London,
CHARLES GORDON, M.D., Boston, Massachusetts, U.S.,
THOMAS GRAHAM, F.R.S., Professor of Chemistry, University College, London,
JOHN GRAHAM, M.D., Brampton, near Carlisle,
R. J. GRAVES, M.D., M.R.I.A., Physician to the Meath Hospital, Dublin,
G. T. GREAM, Esq., Surgeon to the Queen Charlotte Lying-in Hospital, London,
HORACE GREEN, A.M., M.D., Vice-President of the Medical Society, New York, U.S.,
WILLIAM ALEX. GREENHILL, M.D., Physician to the Radcliffe Infirmary, Oxford,
WILLIAM AUG. GUY, M.D., Physician to King's College Hospital, London,
JOHN E. HOLBROOK, M.D., Professor in the Medical College, South Carolina, U.S.,
ENOCH HALE, M.D., Physician to the Massachusetts General Hospital, Boston,
ALEX. HARVEY, M.D., Lecturer at the Marischal College and University of Aberdeen,
CHARLES HASTINGS, M.D., Physician to the Infirmary, Worcester,
JOHN HAVILAND, M.D., Regius Professor of Physic in the University of Cambridge,
JAMES HEYGATE, M.D., F.R.S., Physician to the General Infirmary, Derbyshire,
T. HOBART, M.D., New York, U.S.,
O. W. HOLMES, M.D., Professor of Anatomy and Physiology in the University of Cambridge, New England, and Phys. Massachusetts Gen. Hospital,
THOMAS HODGKIN, M.D., late Lecturer on the Practice of Medicine, St. Thomas's Hospital, Member of the Senate of the University of London,
HENRY HOLLAND, M.D., F.R.S., Physician Extraordinary to the Queen, London,
ALEX. E. HOSACK, M.D., New York, U.S.,
JAMES JACKSON, M.D., Emeritus Professor of the Theory and Practice of Physic in the University of Cambridge, U.S.,
JAMES JAMIESON, M.D., Aberdeen,
JOHN JEFFRIES, M.D., Consulting Physician to Massachusetts General Hospital, U.S.,
GEO. SAMUEL JENKS, M.D., Consulting Physician to the Dispensary, Brighton,
GEORGE JOHNSON, M.D., Assistant Physician to the King's College Hospital,
BENCE JONES, M.D., F.R.S., Physician, St. George's Hospital, London,
GEORGE HAYNES JONES, M.D., Ashling House, Hants,
WHARTON JONES, Esq. F.R.S. Prof. of Anat. and Physiol. in the Charing Cross Hosp.,
GEORGE KENNION, M.D., Physician to the Hospital, Harrowgate,

WM. CHAS. KERR, M.D., Physician to the Infirmary, Northampton,
CHARLES ASTON KEY, Esq., F.R.S., F.R.C.S., Senior Surgeon, Guy's Hospital,
JOHN KIDD, M.D., F.R.S., Regius Professor of Physic in the University of Oxford,
SIR ARNOLD T. KNIGHT, M.D., Liverpool,
JAMES LAURIE, M.D., Surgeon to the Infirmary, Glasgow,
THOMAS LAYCOCK, M.D., Lecturer on the Theory and Practice of Medicine in the York Medical School, York,
HENRY LEE, M.D., Physician to the Farringdon Dispensary, London,
THOMAS STAFFORD LEE, Esq., M.R.C.S., London,
JOHN C. W. LEVER, M.D., Lecturer on Midwifery at Guy's Hospital,
Sir JOHN LIDDELL, M.D., F.R.S., Inspector of Hospitals, Phys. to Greenwich Hosp.,
WM. JOHN LITTLE, M.D., Physician to the London Hospital,
ALEXANDER J. LIZARS, M.D., Prof. of Anatomy, Marischal Col. and Univ., Aberdeen,
CHARLES LOCOCK, M.D., First Physician-Accoucheur to the Queen,
JOHN M. MACFARLANE, M.D., Surgeon to the Infirmary, Glasgow,
SIR JAMES M'GRIGOR, BART., M.D., F.R.S., Director Gen. of the Army Med. Depart.,
WM. MACINTYRE, M.D., Phys. to the Metropolitan Convalescent Institution, London,
ALEXANDER MACKINNON, M.D., Aberdeen,
JAMES MACKNESS, M.D., Hastings,
JOHN MACROBIN, M.D., Prof. of Medicine, Marischal College and Univ. Aberdeen,
J. O. M'WILLIAM, M.D., F.R.S., Surgeon to the Board of Customs, London,
W. H. MADDEN, M.D., Physician to the Dispensary, Torquay, Devon,
ALDEN MARCH, M.D., Professor of Surgery, Albany Medical College, U.S.,
SIR HENRY MARSH, BART., M.D., President of the College of Physicians, Dublin,
HENRY MARSHALL, M.D., F.R.S., Deputy Inspector of Army Hospitals, Edinburgh,
G. ANNE MARTIN, M.D., Ventnor, Isle of Wight,
JAMES RANALD MARTIN, Esq., F.R.S., F.R.C.S., London,
PETER MARTIN, Esq., M.R.C.S., Reigate, Surrey,
JAMES MARTIN, Esq., Assistant-Surgeon, R.N.,
GEORGE MAY, Esq., F.R.C.S., Senior Surgeon to the Royal Berks Hospital, Reading,
CHARLES D. MEIGS, M.D., Professor of Midwifery in Jefferson College, Philadelphia, U.S.,
JAMES MILLER, Esq., Professor of Surgery in the University of Edinburgh, Surgeon to the Queen for Scotland,
JOSEPH MOORE, M.D., Physician to the Freemasons Royal Female Charity, London,
GEORGE MOORE, M.D., Hastings,
THOMAS MORTON, Esq., F.R.C.S., Assistant Surgeon to Univ. Col. Hospital, London,
JAMES MOULTRIE, M.D., Professor in the Medical College, South Carolina, U.S.,
EDWARD WM. MURPHY, M.D., Professor of Midwifery in University College, London,
THOMAS D. MÜTTER, M.D., Professor of Surgery in Jefferson College, Philadelphia,
WILLIAM NEWNHAM, Esq., M.R.C.S., Farnham, Surrey,
GEORGE NEWPORT, Esq., F.R.S., F.L.S., F.R.C.S., London,
DANIEL NOBLE, Esq., M.R.C.S., Manchester,
JOHN NORTH, Esq., F.L.S., F.R.C.S., formerly Lecturer on Midwifery, Middlesex Hospital, London,
GEORGE OGILVIE, M.D., Lecturer at King's College and University, Aberdeen,
JOHN F. OGILVIE, M.D., Aberdeen,
FRANCIS OGSTON, M.D., Lecturer, Marischal College and University, Aberdeen,
DREWRY OTTLEY, Esq., M.R.C.S., London,
BENJAMIN FONSECA OUTRAM, M.D., F.R.S., Inspector of Naval Hospitals, London,

RICHARD OWEN, Esq., F.R.S., Hunterian Professor, Royal College of Surgeons, Lond.,
G. E. PAGET, M.D., Physician to Addenbrooke's Hospital, Cambridge,
THOMAS PAGET, Esq., F.R.C.S., Surgeon to the Infirmary, Leicester,
JAMES PAGET, Esq., F.R.C.S., Professor of Anatomy to the Royal College of Surgeons, Assistant Surgeon to St. Bartholomew's Hospital,
CHARLES L. PARKER, Esq., F.R.C.S., Surgeon to the Radcliffe Infirmary, Oxford,
EDMUND A. PARKES, M.D., Assistant Physician to Univ. College Hospital, London,
GEORGE PARKMAN, M.D., Boston, U.S.,
JOHN PAUL, M.D., Elgin,
THOMAS B. PEACOCK, M.D., Physician to the Royal Free Hospital, London,
ABEL L. PEIRSON, M.D., Salem, Massachusetts, U.S.,
JONATHAN PEREIRA, M.D., F.R.S., Physician to the London Hospital, Examiner in Materia Medica in the University of London,
BENJAMIN PHILLIPS, Esq., F.R.S., F.R.C.S., Surg. to the Westminster Hosp., Lond.,
GEORGE PILCHER, Esq., F.R.C.S., Lecturer on Surgery, London,
JOHN POPHAM, M.D., Cork,
JAMES COWLES PRICHARD, M.D., F.R.S., Member of the Institute of France, Lond.,
WILLIAM PROUT, M.D., F.R.S., London,
RICHARD QUAIN, Esq., F.R.S., Professor of Anatomy in University College, London,
RICHARD QUAIN, M.D., Assistant Physician to the Hospital for Consumption, London,
FRANCIS HENRY RAMSBOTHAM, M.D., Phys. to the Royal Maternity Charity, Lond.,
GEORGE RAINY, Esq., Lecturer, King's College and University, Aberdeen,
JOHN REID, M.D., F.R.C.P.E., Professor of Anatomy in the University of St. Andrews,
JOHN REID, Esq., Assistant-Surgeon, R.N.,
ARCHIBALD ROBERTSON, M.D., F.R.S., Phys. to the General Infirmary, Northampton,
JAMES ROBINSON, Esq., London,
J. KEARNEY ROGERS, M.D., Surgeon to the New York Hospital, U.S.,
J. FORBES ROYLE, M.D., F.R.S., Professor of Materia Medica, King's College, London,
F. F. SANKEY, M.D., R.N., Malta,
EDWIN SAUNDERS, Esq., M.R.C.S,, Surgeon Dentist, St. Thomas's Hospital,
HENRY SAVAGE, Esq., M.D., F.R.C.S., London,
JOHN SCOTT, M.D., Examining Physician to the Honourable E. I. C., London,
JOHN SCOTT, M.D., Fellow of the Royal College of Physicians, Physician in Ordinary to the Queen for Scotland,
EDWARD SCHOLEFIELD, M.D., Physician to the Dispensary, Doncaster,
THOMAS SHAPTER, M.D., Physician to the Devon and Exeter Hospital,
WILLIAM SHARPEY, M.D., F.R.S., Prof. of Physiology, University College, London,
GEORGE SHATTUCK, M.D., Consulting Physician, Massachusetts General Hospital, U.S.,
CHARLES SHEPHERD, M.D., Professor in the Medical College, South Carolina, U.S.,
EDWARD SIEVEKING, M.D., Physician to the Northern Dispensary, London,
JAMES Y. SIMPSON, M.D., Professor of Midwifery in the University of Edinburgh,
JOHN SNOW, M.D., Lecturer on Medical Jurisprudence, Aldersgate School of Medicine,
JOHN SODEN, Esq., F.R.C.S., Surgeon to the Bath General Hospital,
SAMUEL SOLLY, Esq., F.R.S., F.R.C.S., Assistant Surgeon to St. Thomas's Hospital,
JOHN SPALDING, Esq., London,
JAMES SPIERS, M.D., Greenock,
GEORGE JAMES SQUIBB, Esq., M.R.C.S., London,
R. A. STAFFORD, Esq., F.R.C.S., Surgeon to the Marylebone Infirmary, London,

EDWARD STANLEY, Esq., F.R.S., Vice-President of the Royal College of Surgeons, Surgeon to St. Bartholomew's Hospital,

JOHN STIRLING, M.D., R.N.,

WILLIAM STOKES, M.D., M.R.I.A., Physician to the Meath Hospital, Dublin,

SIGISMUND STOLTERFORTH, M.D., Senior Physician to the Dispensary, Dover,

ROBERT J. A. STREETEN, M.D., Physician to the Infirmary, Worcester,

PETER SUTHER, M.D., R. N., Royal Dockyard, Woolwich,

A. J. SUTHERLAND, M.D., F.R.S., Physician to St. Luke's Hospital, London,

JOHN SUTHERLAND, M.D., Physician to the Dispensary, Liverpool,

W. E. SWAINE, M.D., Physician Extraordinary to H. R. H. the Duchess of Kent, Lond.,

JOHN ADDINGTON SYMONDS, M.D., Consulting Physician, General Hospital, Bristol,

JOHN TAYLOR, M.D., Physician to the Infirmary, Huddersfield,

JOHN TAYLOR, M.D., Fellow of the Royal College of Physicians, Edinburgh,

HIBBERT TAYLOR, M.D., Surgeon to the Eye Dispensary, Liverpool,

ALFRED S. TAYLOR, F.R.S., Lecturer on Medical Jurisprudence at Guy's Hospital, Lond.,

T. P. TEALE, Esq., F.R.C.S., Surgeon to the General Hospital, Leeds,

WILLIAM THOMPSON, M.D., Bognor, Sussex,

ANTHONY TODD THOMSON, M.D., Professor of Materia Medica and of Medical Jurisprudence, University College, London,

ROBERT DUNDAS THOMSON, M.D., Andersonian Lecturer on Chemistry, Glasgow,

THOMAS THOMSON, M.D., Physician to the Infirmary, Stratford-on-Avon,

SPENCER THOMSON, M.D., Haunton, Burton-on-Trent,

THOMAS THOMSON, M.D., Inverary,

JOHN THURNAM, M.D., Medical Superintendent of the Retreat, York,

J. NEWTON TOMKINS, Esq., F.R.C.S., National Vaccine Establishment, London,

JOSEPH TOYNBEE, Esq., F.R.S., F.R.C.S., Senior Surgeon to the St. George's and St. James's Dispensary,

JAMES TRAIL, M.D., Monymusk,

SAMUEL TUKE, Esq., York,

NICHOLAS TYACKE, M.D., Physician to the Infirmary, Chichester,

JAMES VOSE, M.D., Liverpool,

CHARLES WARD, Esq., M.R.C.S., Doncaster,

WALTER HAYLE WALSHE, M.D., Prof. of Clinical Medicine, University College, Physician to the Hospital for Consumption, London,

JOHN WARE, M.D., Professor of the Principles and Practice of Medicine in the University of Cambridge, U.S.,

JOHN E. WARREN, M.D., Emeritus Professor of Anatomy and Surgery in the University of Cambridge, U.S.,

J. MASON WARREN, M.D., Surgeon to the Massachusetts General Hospital, U.S.,

WILLIAM C. WATT, M.D., R.N.,

THOMAS WATSON, M.D., F.R.S., late Professor of Medicine in King's College, London,

THOMAS SPENCER WELLS, Esq., Surgeon, R.N.,

CHARLES WEST, M.D., Lecturer on Midwifery, Middlesex Hospital,

DAVID B. WHITE, M.D., Newcastle-on-Tyne,

JOSHUA WHITRIDGE, M.D., Charleston, U.S.,

GEORGE WILKES, M.D., Surgeon to the New York Eye Infirmary, U.S.,

CALEB WILLIAMS, Esq., Lecturer on Materia Medica, &c., York Medical School,

CHARLES J. B. WILLIAMS, M.D., F.R.S., Prof. of Medicine in Univ. Coll., London,

BENJAMIN WILLIAMSON, M.D., Aberdeen,

JOSEPH WILLIAMSON, M.D., Aberdeen,
L. R. WILLAN, M.B., Penzance, Cornwall,
AYLMER WILSON, M.D., Edinburgh,
ERASMUS WILSON, Esq., F.R.S., Consulting Surgeon to the St. Pancras Infirmary,
JOHN WILSON, M.D., F.R.S., Inspector of Hospitals, R.N.,
HON. ROBERT C. WINTHORP, Speaker of the United States House of Representatives at Washington, U.S.,
JAMES WOOD, M.D., Fellow of the Royal College of Physicians, Edinburgh,
JAMES WOODMAN, M.D., Chichester,
SAMUEL WRIGHT, M.D., Physician to Queen's Hospital, and Professor of Clinical Medicine in Queen's College, Birmingham;

WHO

(TO BORROW THEIR OWN FLATTERING EXPRESSIONS)

"TO TESTIFY THEIR RESPECT FOR HIS LITERARY AND SCIENTIFIC CHARACTER,
"AND THEIR SENSE OF THE VALUE OF HIS DISINTERESTED SERVICES IN
"ESTABLISHING AND CARRYING ON, FOR A PERIOD OF TWELVE YEARS, THE
"BRITISH AND FOREIGN MEDICAL REVIEW,"

HAVE ASSOCIATED TO PRESENT HIM

WITH

"A LASTING MEMORIAL OF THEIR APPROVAL AND ESTEEM;'

THE WORK

TO WHICH THE PRESENT VOLUME IS AN INDEX,

IS,

WITH A DEEP SENSE OF THE HONOUR CONFERRED UPON HIM,

MOST RESPECTFULLY AND MOST GRATEFULLY,

Dedicated

BY THEIR OBEDIENT HUMBLE SERVANT

JOHN FORBES.

